# Arabic Version of the Family Health Climate Questionnaire in a Saudi Population: Translation, Validation, and Reliability

**DOI:** 10.3390/nu18142397

**Published:** 2026-07-22

**Authors:** Israa M. Shatwan, Noha M. Almoraie, Najlaa M. Aljefree

**Affiliations:** Food and Nutrition Department, Faculty of Human Sciences and Design, King Abdulaziz University, Jeddah 3270, Saudi Arabia; nalmorie@kau.edu.sa (N.M.A.); naljefree@kau.edu.sa (N.M.A.)

**Keywords:** family health climate, validation, nutrition, physical activity, Arabic

## Abstract

**Background/Objectives**: The family health climate (FHC) questionnaire comprises two subscales: physical activity (FHC-PA) and nutrition (FHC-NU). We aimed to translate the FHC into Arabic, culturally adapt it, and evaluate its reliability and validity among adult Arabic speakers. **Methods**: The original scale was translated and back-translated, and face and content validity of the scale was reviewed by 10 nutritionists. The scale reliability was measured using Cronbach’s alpha (α). Exploratory factor analysis was conducted to establish construct validity. **Results**: The FHC demonstrated an acceptable level of content validity, with a scale content validity index/average of 0.93 and index/universal of 0.51. The scale exhibited high internal consistency (Cronbach’s alpha = 0.956, 0.928, and 0.939 for the total FHC, FHC-PA, and FHC-NU, respectively). Strong Kaiser–Meyer–Olkin values for FHC-PA and FHC-NU (0.946 and 0.947, respectively) and a significant Bartlett’s test of sphericity (*p* < 0.001) were observed. For the FHC-PA, a three-factor structure was identified, explaining 51.5% of the variance, whereas the FHC-NU exhibited a four-factor structure explaining 49.2% of the variance. **Conclusions**: The Arabic FHC scale is a valid and reliable tool for evaluating the family environment that influences health-related behaviors. It can serve as a valuable resource for developing family-oriented strategies for health promotion and disease prevention.

## 1. Introduction

The family is the foundation of individual health and development, as well as the cornerstone of the community [1]. The family’s ecocultural pathway encompasses shared values and goals and daily habits (including routines around meals, choice and preparation of food, joint physical activities, risk behaviors, and social engagement), along with environmental factors such as income, education, and resources, all of which are important in promoting health [2]. Family health is defined as “a resource at the level of the family unit that develops from the intersection of the health of each family member, their interactions and capacities, as well as the family’s physical, social, emotional, economic, and medical resources” [3]. Formal and informal family rules, along with health routines, are shaped as family members share their understanding, opinions, and behaviors, which in turn influence individuals’ health behavior patterns [4].

Family function is reflected in aspects such as communication patterns, role fulfillment, adaptability, and behavioral control, and is associated with healthier outcomes among adolescents, including a lower risk of overweight and obesity, reduced sedentary behavior, decreased fast-food consumption, higher intake of fruits and vegetables, sharing family meals and daily breakfast, and improved sleep duration [5,6]. Moreover, family function has been associated with mental health and healthy behavior [7,8]. Family health care provided to members across life stages is more economically valuable than that provided by the medical system [9]. Family-level factors typically considered include household composition, family structure, occasionally income, and the health of individual family members [10].

Family health climate (FHC) is an example of a family-level factor, reflecting how daily household rituals and interaction between family members shape health behaviors and perceptions. These interactions occur frequently over an extended period and create a “climate” that represents a core aspect of family interrelationships and the family environment. FHC captures shared perceptions and cognitions concerning health and health behaviors among family members [11]. First defined by Niermann et al. [11], the framework covers routine health habits such as nutrition and physical activity occurring both inside and outside the home. The FHC scales have been tested and validated in diverse populations [12,13] yielding promising results: FHC-PA and FHC-NU scores are associated with individual outcomes, including healthy dietary pattern, physical activity, food parenting style, and children’s body mass index (BMI) [14,15], highlighting the influence of family systems on children’s and adolescents’ health behaviors.

Family serves as the foundational cornerstone of the Saudi Arabian society. Families constitute the individual’s core social network and secure both socioeconomic and psychological stability. The traditional Saudi family is characterized by a patriarchal authority structure that fosters loyalty and solidarity among members, and an extended family plays a significant role in the daily social and emotional integration. Family relationships become particularly important when caregiving is needed to support the well-being and happiness of all the family members. Arabic is spoken by more than 450 million people and holds official status in nearly 25 countries. Therefore, we aimed to translate the FHC questionnaire into Arabic, test the validity of the translated version in adult Arabic speakers, assess the internal consistency among all subscales, and confirm its factor structure.

## 2. Materials and Methods

### 2.1. Study Design

This cross-sectional study was conducted in two phases. Phase one involved translating the questionnaire from the original English version to Arabic after obtaining permission from the developer of the FHC [11]. Phase two involved the evaluation of the validity of the FHC scale in the Saudi Arabian population and testing its psychometric properties. This study was approved by the Biomedical Ethics Research Committee of King Abdulaziz University (reference number 42–25) in accordance with guidelines outlined in the Declaration of Helsinki. Before completing the questionnaire, all participants were required to review the objectives of the study and provide electronic informed consent for participation.

### 2.2. Research Instrument

This scale measures the impact of the family on individual dietary behaviors (FCH-NU) and physical activity levels (FHC-PA). The FHC-PA consists of three subscales (value, cohesion, and information) and 14 items. The FHC-NU comprises four subscales (value, cohesion, communication, and consensus) and 17 items. Each item was given on a four-point rating scale (0 = “definitely false”, 1 = “rather false”, 2 = “rather true”, 3 = “definitely true”), and each response had a score. The items were prefaced with a uniform contextual statement “In our family….”. The total scores for FHC-PA and FHC-NU ranged from 0 to 48 and 0 to 51, respectively, with higher scores reflecting an optimal family health climate.

### 2.3. Cross-Cultural Adaptation Process

The FHC questionnaire was initially conceptualized in German; however, the authors published the English version in their article [11]. This English version was translated into Arabic by five native Arabic-speaking senior researchers in the Food and Nutrition Department, and the translation was checked by two doctors in the department. Subsequently, a back-translation of the Arabic version into English was conducted by three bilingual doctors who are proficient in the English language. This version was then reviewed by two other doctors for comparison with the original version.

### 2.4. Participants

The target population for this study was Saudi adults (aged ≥ 18 years) or native Arabic speakers living in Saudi Arabia. Literate, healthy adults of both sexes were invited to participate. Participants were recruited through social media platforms such as WhatsApp, X, and Telegram and e-mail. The recruitment was conducted between March and May 2025. Participants were required to provide their email address to avoid duplicated participation.

### 2.5. Sample Size

The study utilized a 10:1 item-to-response ratio as the recommended sample size for the validation phase, resulting in a required total of 310 participants [16]. Moreover, an a priori sample size estimation was applied, resulting in the requirement of 100 participants for the structural equation modeling technique to run factor analysis [17]. Therefore, the minimum number of participants required to adequately power our analysis was 410 participants.

### 2.6. Validation Process

After the translation process, the questionnaire was redistributed to 10 nutritionists to test validity (face validity, floor and ceiling effects, and content validity) [18].

#### 2.6.1. Face Validity

Validity testing involved the measurement of three main aspects: completeness of content, comprehensibility, and time required. A group of 10 experts was involved in this phase. First, they were asked if the questionnaire covered the most important aspect of family health climate; if the answer was “No,” the respondent was asked to indicate “which aspects they would incorporate”. Second, the experts were asked to indicate which questions required enhancement to improve clarity. Lastly, they evaluated the time needed to complete the questionnaire on a scale of 0–10, with 10 corresponding to “completely okay.”

#### 2.6.2. Floor and Ceiling Effect

Ceiling and floor effects occurred when a high frequency of participants achieved the maximum or minimum possible score, which caused non-normal distribution and skewed frequencies at the scale’s boundaries. The floor and ceiling effects of the questionnaire were assessed across all items using the total scores. Ceiling or floor effects were determined if more than 15% of responses scored at the absolute minimum or maximum.

#### 2.6.3. Content Validity

Expert panelists assessed the content validity, i.e., the relevance, of the translated FHC questionnaire items using a four-point Likert Scale from 1 (not relevant) to 4 (is very relevant). Scores of 3 and 4 were recorded as 1 (relevant), while scores of 1 and 2 were recorded as 0 (not relevant). The content validity index (CVI) was calculated using an average scale. The CVI for individual items (I-CVI) and for the scale (S-CVI) was determined by a panel of 10 experts reviewing each question. The relevance of each item was rated on a 5-point Likert scale (1 = not at all relevant to 5 = very relevant). Items with scores of 4 and 5 were categorized as relevant. The SCVI/average and S-CVI/universal agreements were computed following established guidelines [19].

### 2.7. Pilot Testing

Prior to distributing the final version of the scale, interviews were conducted with 20 adult participants. The participants provided feedback on the formulation of each item, indicating whether the statements were clear, confusing, or easy to answer. Pilot testing was conducted by distributing the questionnaires online, and comments were provided regarding any confusion or misunderstanding of any item.

### 2.8. Data Collection

The participants completed the questionnaire electronically using Google Forms. The first part of the questionnaire consisted of questions to collect sociodemographic data including age, sex, marital status, income level, educational level, smoking status, parents’ education, parents’ job, number of family members, number of years family formed, participants’ position in the family, number of meals the family eats together, type of meals, self-reported height and weight, and physical activity levels. The second part was the FHC scale.

### 2.9. Internal Consistency Reliability

The internal consistency of the research instrument was evaluated utilizing Cronbach’s alpha. An alpha value of 0.70 or higher was considered satisfactory for establishing instrument reliability [20].

### 2.10. Statistical Analysis

The data obtained were analyzed using SPSS (version 29.0; SPSS Inc., Chicago, IL, USA), and are presented using means and standard deviations for quantitative data, while categorical variables are expressed as frequencies and percentages. The internal consistency of the FHC was assessed using Cronbach’s alpha. Spearman’s correlation coefficients among the items were calculated to assess correlation between measured concepts and continuous variables (age and BMI). The construct validity of the FHC scale was assessed using exploratory factor analysis (EFA) and Kaiser–Meyer–Olkin (KMO) measurements of sampling adequacy. Bartlett’s test of sphericity was used to assess the factorability of the FHC-PA and FHC-NU separately. EFA was carried out on both FHC-PA and FHC-NU to identify the underlying factor structures using principal axis factor extraction with oblique Promax rotation. The number of factors was extracted based on an initial eigenvalue cutoff of 0.80, to retain factors that have meaningful variance contributions without inflating the model and the original FHC factor model [21]. Factor loadings ≥ 0.30 were considered significant for item retention. An independent *t*-test and one-way ANOVA were used to examine association between total FHCA and its subscales’ scores, and sociodemographics of participants. A *p* value ≤ 0.05 was considered significant.

## 3. Results

### 3.1. Face Validity and Content Validity

The findings regarding face validity are presented in Table 1. Nutritional experts and faculty members of the Food and Nutrition Department agreed that the FHC questionnaire was complete (100%). Overall, 70% of the experts rated the statements of the FHC as comprehensible. The experts reported a high similarity in the statements, especially between each subsection (FHC-PA and FHC-NU), and some sentences were repeated. Consequently, to increase the readability of statements, we highlighted the verb in each statement to make it distinguishable. A suggestion was made to replace some statements with others, for example, decrease watching time and increase activity; however, this was not implemented to preserve the integrity of the original content. Feedback on questionnaire completion time was satisfactory, with a mean of 10 out of 10 responses. The values of SCVI/Ave and S-CVI/UA were calculated to be 0.93 and 0.51, respectively. The mean percentage of experts was 0.93 (Table 2).

### 3.2. Characteristics of Study Participants

Table 3 presents the characteristics of the participants. The mean age was 31.0 ± 9.8 years, and the mean BMI was 25.2 ± 5.3 kg/m^2^. Female participants comprised 69.3% of the sample, and 30.7% were male. Furthermore, 54.6% of participants were unmarried and 45.4% were married. Overall, 68.6% of participants had a university degree, 19% had a postgraduate degree, and 12.5% had only completed high school. Approximately half of the participants (48.9%) were employed, 26% were students, and 23.4% unemployed. The majority, 86.3%, were non-smokers, and 45.2% were lightly active. Most parents held high school degrees, with 68.7% of mothers being unemployed and 51.1% of fathers retired. Furthermore, 45.5% of families had 4–6 members and 41.2% had 7 members. In addition, 73.3% had a family duration of >10 years, while 23.8% had a family duration between 1 and 10 years. Moreover, 41.0% of the participants who completed the questionnaires were daughters, and 27.1% were mothers, while fathers and sons represent 12.8% and 19.1%, respectively. Additionally, 48.9% of families ate two meals together, while 24.3% ate only one meal together. A total of 33.6% had a monthly household income between 10,000 and 20,000 SAR. The mean and standard deviation of the FHC and its subscales, the FHC-PA and FHC-NU, are presented in Table 4.

### 3.3. Reliability Analysis

In total, 786 participants were included in the reliability analysis. Cronbach’s alpha for the total FHC was 0.956, which confirmed strong internal consistency (Table 5). Subscales FHC-PA and FHC-NU also had excellent internal consistency with Cronbach’s α = 0.928 and 0.939, respectively.

### 3.4. Factor Analysis

**Figure 1:**
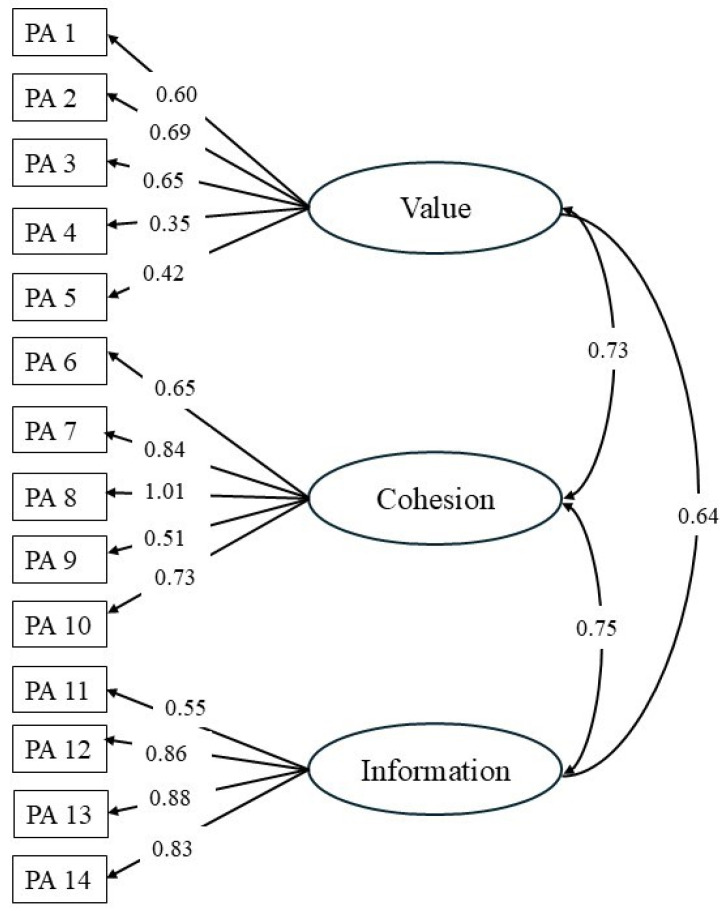
The models with factor loadings and correlations for family health climate—physical activity.

**Figure 2:**
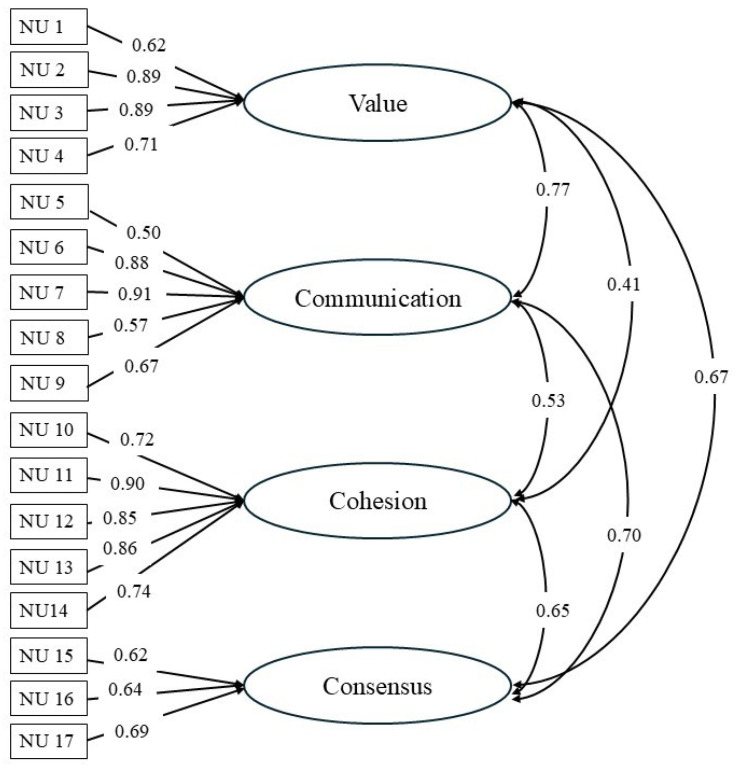
The models with factor loadings and correlations for family health climate—nutrition.

Exploratory factor loading analysis was conducted separately for FHC-PA and FHC-NU, identifying three factors for FHC-PA and four factors for FHC-NU. Results from the KMO measure of sampling adequacy for FHC-PA and FHC-NU (=0.946 and 0.947, respectively) and Bartlett’s test of sphericity (χ^2^ = 7081.3 (91), *p* < 0.001 and χ^2^ = 9601.0 (136), *p* < 0.001, respectively) indicated that data for both scales were suitable for conducting factor analysis (Table 6). The three dimensions of the FHC-PA scale explained 51.5% of the variance and the four dimensions of the FHC-NU scale explained 49.2%. The models with factor loadings and correlations are shown in Figure 1 and Figure 2.

Table 7 shows the results of Bivariate Pearson’s correlations among the total FHC, its subscales, age, and BMI of the study participants. FHC, FHC-PA, and FHC-NU positively correlated with each other. Age was positively associated with the FHC-PA and FHC-NU levels (*p* < 0.01 and 0.05, respectively). However, BMI was not associated with the total FHC, FHC-PA, and FHC-NU levels.

Table 8 shows the association between the total FHC and its subscales, and sex, marital status, and education of the study participants. Female participants had higher FHC-PA compared with male participants (*p* = 0.012). Married participants had higher total FHC and subscale scores than unmarried participants (*p* < 0.001). We also noted a significant trend of decreasing total FHC and subscale scores with increasing education level.

## 4. Discussion

In this study, we aimed to translate and evaluate the validity and reliability of the Arabic version of the FHC in an Arab population. The findings showed that the translation, validity, and reliability of the FHC tool achieved satisfactory scores based on evaluations performed by a panel of expert nutritionists. The FHC was first developed by Niermann et al. [11] to build a qualitative approach to explore how families manage their daily health-related routines, as family cohesion encourages members to eat healthy foods and be physically active. Family is a well-known key predictor of an individual’s health status, because it is where habits are built from the earliest age [22]. Therefore, it is useful to translate the FHC into Arabic to assist researchers in investigating how health-related habits among Arab families are involved in increasing the risk of non-communicable diseases.

Given that the original FHC scale was in German and English, it is necessary to test the accuracy of the Arabic translation because interpretation inevitably reflects a particular understanding of a text, and cultural differences impact encoding and decoding [23]. For example, two statements in the FHC-PA section on cohesion factors—“we like being together during physical activities” and “we have fun doing physical activities together”—give highly similar meanings in Arabic language; thus, based on panel feedback, a modification of the verb used was applied. Another change made was in the statement “we read newspaper or magazine articles on fitness, physical activity, and exercise”; because social media has replaced traditional sources like newspapers [24,25], changes were made accordingly to “following experts on social media”. Both the original and adapted items assess information on consumption behavior and engagement with written/textual media; the transition from traditional to digital platforms represents a change in delivery mechanism while maintaining conceptual equivalence with the original measure. Notably, the overall scale structure and factor composition remained comparable because scores were analyzed at the scale level rather than the item level.

The final items in the FHC scale showed good validity and reliability. The FHC scale was rated high for its clarity and comprehensibility and was completed by an expert panel in face validity. Two values were calculated for the content validity: SCVI/Ave and SCVI/UA. The SCVI/Ave method suggested that the overall content validity of FHC was high (SCVI/Ave = 0.93), whereas the SCVI/UA approach showed a fair level (SCVI/UA = 0.51). The numerator SCVI/Ave will always be greater than SCVI/UA because the likelihood of chance universal agreement around each I-CVI value equal to 1.00 decreases when the number of experts increases [19]. The results of the SCVI/UA in our study were considered fair according to the recommendation given by Polit & Beck [26] and similar to other studies that conducted Arabic translation and validation of other nutrition-related scales [27,28]. Cronbach’s alpha for the Arabic FHC was 0.9, indicating a very high acceptable internal consistency.

The results of the factor loading analysis of the FHC-PA and FHC-NU demonstrated significant agreement with the original FHC scales. All three original factors (value, cohesion, and information) of the FHC-PA were replicated using the Arabic version. The original four factors of the FHC-NU scale (value, communication, cohesion, and consensus) were replicated. In our study, female participants had higher FHC-PA scores, similarly to a cross-sectional study reporting that female participants scored higher in fitness than male participants [29]. This can be explained by the increasing interest among women, especially young women, in increasing physical activity to enhance their health. In addition, findings from this study indicated that married and less educated participants had higher FHC, FHC-PA, and FHC-NU scores. Therefore, family structure serves as an important determinant of health outcomes; families that maintain a stable relationship and positive environment exhibited better health outcomes among their members [30,31]. In contrast with our findings, a previous study reported that higher education is associated with higher FHC scores [32]; this may be because people with lower education levels tend to prioritize sharing time with their families, which creates a shared, supportive cultural environment.

This study had certain limitations. Recall bias may have occurred because of the self-reporting nature of the questionnaire. As Saudi participants were dominant in our study sample, further validation studies are needed to confirm the robustness of the Arabic FHC scale in other Arab contexts. The absence of confirmatory factor analysis limits our ability to confirm the factor structure’s replicability; therefore, future researchers are encouraged to conduct confirmatory factor analysis with access to independent larger samples to strengthen the evidence base for this instrument’s validity. The cross-sectional study design is another limitation because it yields weaker evidence for causality. The convenience sampling approach may have also introduced sampling bias because it relies on availability and willingness; thus, the sample only includes individual who are easily accessible and chose to respond. Finally, self-reported anthropometric data may introduce measurement errors and reporting biases; however, it was not a main factor in our validation study.

## 5. Conclusions

This study provides a psychometrically robust Arabic version of the FHC scale, suitable for assessing families’ daily routines related to nutrition and physical activity within Arabic cultural contexts. The scale offers substantial research utility, enabling investigation of how family climate (eating behavior and physical activity) affects disease incidence and prevention, and supporting the development of strategies to promote healthy lifestyles among communities. The findings demonstrated associations of sex, marital status, and education with total FHC, FHC-PA and FHC-NU. Further studies are needed to examine the validity and reliability of this scale in other Arabic-speaking countries to confirm these results.

## Figures and Tables

**Table 1:** Face validity items.

Item	Result
Completeness of contents	100%
Comprehensibility	70%
Appropriateness of time complete (mean ± SD)	10 ± 0

**Table 2:** Content validity.

Item	Result
Scale content validity index/universal	0.51
Scale content validity index/average	0.93
Average proportion	0.93

**Table 3:** Characteristics of study participants, n = 786.

Sociodemographic Parameter	Frequency n (%)
Sex	
Male	241 (30.7)
Female	545 (69.3)
Marital status	
Married	357 (45.4)
Unmarried	429 (54.6)
Education	
High school	98 (12.5)
University	539 (68.6)
Postgraduate	149 (19.0)
Occupation	
Student	204 (26.0)
Employed	384 (48.9)
Unemployed	187 (23.8)
Retired	11 (1.4)
Smoking	
Non-smoker	678 (86.3)
Smoker	73 (9.3)
Past smoker	35 (4.5)
Mother education	
High school	422 (53.7)
University	303 (38.5)
Postgraduate	61 (7.8)
Mother occupation	
Employed	177 (22.5)
Unemployed	540 (68.7)
Retired	54 (6.9)
Own business	15 (1.9)
Father education	
High school	370 (47.1)
University	320 (40.7)
Postgraduate	96 (12.2)
Father occupation	
Employed	280 (35.6)
Unemployed	47 (6.0)
Retired	402 (51.1)
Own business	57 (7.3)
Family size	
1–3 members	104 (13.2)
4–6 members	358 (45.5)
7 or more	324 (41.2)
Family formation duration	
Less than 1 year	23 (2.9)
1–5 years	89 (11.3)
6–10 years	98 (12.5)
More than 10 years	576 (73.3)
Role of participants who filled in the survey in the family	
Father	101 (12.8)
Mother	213 (27.1)
Son	150 (19.1)
Daughter	322 (41.0)
Number of meals eaten together in the family	
None	27 (3.4)
One meal	191 (24.3)
Two meals	384 (48.9)
Three meals	184 (23.4)
Physical activity	
Sedentary	210 (26.7)
Light	355 (45.2)
Moderate	175 (22.3)
Vigorous	46 (5.9)
Family income	
Less than 5000 SAR	86 (10.9)
5000–10,000 SAR	247 (31.4)
10000–20,000 SAR	264 (33.6)
More than 20,000 SAR	189 (24.0)

3.75 SAR is equal to 1 US dollar.

**Table 4:** Descriptive analysis of family health climate scale and its subscale scores.

Scale	Mean (SD) Score
FHC	67.1 (16.7)
FHC-PA	27.7 (8.9)
FHC-NU	39.3 (9.1)

**Table 5:** The reliability analysis of FHC-Arabic and its subscales.

	Number of Items	Cronbach’s α
Family health climate	31	0.956
FHC-PA	14	0.928
FHC-NU	17	0.939

**Table 6:** Kaiser–Meyer–Olkin measure and Bartlett’s test.

FHC-PA		
Item		Value
Kaiser–Meyer–Olkin measure of sampling adequacy of data		0.946
Bartlett’s test of sphericity	Approx. chi-square	7081.3
	Df	91
	Sig.	<0.001
**FHC-NU**		
Kaiser–Meyer–Olkin measure of sampling adequacy of data		0.947
Bartlett’s test of sphericity	Approx. chi-square	9601.0
	Df	136
	Sig.	<0.001

**Table 7:** Bivariate correlations between measured FHC scores and other continuous variables.

	FHC-PA	FHC-NU	FHC Total	Age
FHC-PA	1			
FHC-NU	0.704 **	1		
FHC total	0.921 **	0.925 **	1	
Age	0.133 **	0.089 *	0.012	1
Body mass index	0.002	−0.019	−0.009	0.357 **

** Correlation is significant at the 0.01 level (2-tailed). * Correlation is significant at the 0.05 level (2-tailed).

**Table 8:** Association between measured FHC scores and participants’ sociodemographic parameters.

Sociodemographic	FHC-PA	FHC-NU	FHC Total
Sex *			
Male	27.2 ± 9.1	39.1 ± 9.3	66.3 ± 17.1
Female	28.9 ± 8.5	39.9 ± 8.7	68.9 ± 15.8
*p* value	0.012	0.212	0.047
Marital status *			
Married	29.2 ± 8.1	41.0 ± 7.7	70.2 ± 14.3
Unmarried	26.6 ± 9.4	37.9 ± 10.0	64.5 ± 18.1
*p* value	<0.001	<0.001	<0.001
Education **			
High school	30.0 ± 8.4	41.0 ± 8.9	71.1 ± 16.4
University	28.3 ± 8.7	39.5 ± 9.2	67.8 ± 16.5
Postgraduate	24.4 ± 9.1	37.4 ± 9.0	61.9 ± 16.5
*p* value	<0.001	0.007	<0.001

Data presented as mean ± standard deviation. * *p* value calculated using independent *t*-test; ** *p* value calculated using one-way ANOVA. *p* value < 0.05 considered significant.

## Data Availability

The original contributions presented in this study are included in the article/Supplementary Material. Further inquiries can be directed to the corresponding author.

## Supplementary Materials

The following supporting information can be downloaded at: https://www.mdpi.com/article/10.3390/nu18142397/s1.

## Abbreviations

The following abbreviations are used in this manuscript:

FHC	Family health climate
FHC-PA	Family health climate—physical activity
FHC-NU	Family health climate—nutrition
BMI	Body mass index
CVI	Content validity index
S-CVI	Scale content validity index
KMO	Kaiser–Meyer–Olkin
